# Phosphorus-Containing Telomers as UV-Curable Binders of Solvent-Free Varnish Coatings

**DOI:** 10.3390/ma15248991

**Published:** 2022-12-16

**Authors:** Agata Kraśkiewicz, Agnieszka Kowalczyk

**Affiliations:** Department of Chemical Organic Technology and Polymeric Materials, Faculty of Chemical Technology and Engineering, West Pomeranian University of Technology in Szczecin, 70-322 Szczecin, Poland

**Keywords:** phosphorus-containing polymers, telomerization, photopolymerization, (meth)acrylates, varnish

## Abstract

The synthesis of novel phosphorus-containing telomers (P-telomers) was conducted via a solution-free UV-initiated telomerization process of butyl acrylate, methyl methacrylate, 2-hydroxyethyl acrylate, and styrene, different phosphorus telogens (dimethyl phosphite (DMPh), dibutyl phosphite (DBPh), diphenyl phosphite (DPPh) or dibutyl phosphate (DBP)), and a radical photoinitiator-acylphosphine oxide (APO). The course of the UV-phototelomerization process was monitored by photo-DSC and the chemical structures of telomers were assessed by FTIR. Final UV-photocurable varnish compositions consisted of prepared P-telomer syrups, crosslinking monomer (pentaerythritol triacrylate; PETIA), and a radical UV-photoinitiator (α-hydroxyalkylphenone, HAP). The influence of P- telomers on the optical and mechanical features of coatings was investigated. Relatively the highest hardness and satisfactory scratch values, as well as water and solvent resistance, were observed for varnish based on DMPh-telomers. While the strongest adhesive bond to a glass substrate was reported for DPPh-telomers. It is worth pointing out that the P-telomers did not affect the gloss values of varnishes in comparison to the telomer-free reference sample.

## 1. Introduction

UV-curable solvent-free coatings are promising for various fields of industrial applications, e.g., electronics [1,2], automotive [3,4], aerospace [5,6], textile industry [7,8], etc. This class of materials draws significant attention principally due to the high-speed curing at room temperature, low emission of organic volatile compounds, and little energy requirements [9,10,11,12]. Binders used in photocurable formulations are mainly based on (meth)acrylates, which are characterized by the high reactivity of polymeric chains and hence wide possibilities of modification [13,14]. Particularly interesting types of (meth)acrylate monomers for coating applications are phosphate (meth)acrylates and poly(meth)acrylates. The presence of phosphorus atoms in the (meth)acrylate structure gives the resulting polymer specific properties, for example, flame retardant [15,16,17], good adhesion to steel substrates [18,19], corrosion-resistant [20,21], biodegradable properties [22,23,24]. An important issue is also the use of phosphate (meth)acrylates in biomedicine, e.g., hydrogels based on copolymers of 2-hydroxyethyl methacrylate and 2-ethylene glycol methacrylate phosphate with potential application as scaffolds for tissue engineering [25]. The common pathway for the synthesis of presented phosphorus-containing monomers is a condensation reaction, phosphorylation, or Pudovik reaction, where the source of phosphorus atoms is, i.e., phosphoryl chlorides, P_2_O_5_, or phosphonic acid esters.

Regarding photocurable compositions based on oligomeric binders containing terminal phosphate or phosphite groups, reports in the literature are limited. Relatively few patents are described. For example, coatings based on polymers with a pendant or terminal phosphorus groups obtained by emulsion/suspension polymerization of (meth)acrylic acid esters and vinyl esters (e.g., styrene, vinyl chloride) with allyl phosphate or phosphoethyl (meth)acrylate [26], polymer composition consisting of suspension polymerization products of unsaturated phosphorus-containing monomers (such as phosphates of hydroxy alkyl (meth)acrylates or dihydrogen phosphate esters of unsaturated alcohols) [27], coating composition compounded of phosphorus acid functional polyol polymers (i.e., unsaturated phosphate esters of polyether glycols, phosphoethyl (meth)acrylate or phosphopropyl (meth)acrylate) [28], coating prepared with photocuring phosphorus-nitrogen acrylic resin [29], and polyurethane-acrylic resin obtained by emulsion polymerization [30]. In contrast, the other invention presents a synthesis of acrylate resins consisting of epoxy acrylates and phosphorus-containing phenol derivatives [31].

The above examples of synthesis of phosphorus-containing polymers are based on emulsion or suspension polymerization (sometimes in an organic solvent). However, an alternative technique preparation of oligomers with phosphorus atoms may be telomerization. Telomerization is a special type of polymerization conducted between one or more polymerizing compounds (taxogens/ monomers; M) and telogen (a molecule with a cleavable bond, e.g., C-H, S-H, P-H, Si-H, also named chain transfer agent; YZ) that leads to the formation of low-molecular-weight products (oligomers/telomers; Y(M)_n_Z) in which the ends of the chains contain fragments derived from telogen breakdown. [32,33]. The formation of telomers is most often initiated by thermal initiators but also using UV-radiation, and γ rays [34,35]. Regarding phosphorus-containing telogens (with P-H groups), they participate mainly in thermally initiated telomerization reactions. The most common telogens suitable for many vinyl monomers are dialkyl phosphites, phosphorus acid, phosphorus tri- and pentachloride, and alkyl dichlorophosphines or dichlorophosphonates. However, thus far, phosphate and phosphonate esters were characterized as less effective chain transfer agents [36]. To the best of the authors’ knowledge, there is no information in the literature about the preparation of phosphorus-containing oligomers/telomers (P-telomers) via a UV-induced process.

This paper presents the novel synthesis of phosphorus-containing (meth)acrylates oligomers (P-telomers) via UV-induced telomerization of n-butyl acrylate, methyl methacrylate, 2-hydroxyethyl acrylate, and styrene in the presence of phosphate or phosphonate esters (as P-telogens) and acylphosphine oxide (as a radical photoinitiator). Prepared phosphorus-containing telomers syrups (P-telomers syrups) were used as binders of UV-curable varnish coatings. Additionally, the varnish compositions contained a crosslinking monomer (pentaerythritol triacrylate) and an additional radical photoinitiator (α-hydroxyalkylphenone). Features of the P-telomers syrups, UV-photocurable varnish compositions, and cured varnishes were analyzed. It was revealed that UV-phototelomerization can be an environmentally acceptable way to produce novel phosphorus-containing binders. Moreover, phosphorus-containing varnish coatings may have potential anti-corrosion, anti-bacterial, and flame-retardant properties and could be applied as protective coatings on glass, metal, or wooden substrates.

## 2. Materials and Methods

### 2.1. Materials

The phosphorus-containing telomers (P-telomers) were synthesized using the following components:

(a) acrylate monomers:

- n-butyl acrylate (BA; BASF, Ludwigshafen, Germany),
- methyl methacrylate (MMA; Sigma Aldrich, Steinheim, Germany),
- 2-hydroxyethyl acrylate (HEA; Across Organics, Geel, Belgium),
- styrene (STY; Sigma Aldrich, Steinheim, Germany).

(b) phosphorus compounds (as telogens)

- dimethyl phosphite (DMPh; Sigma Aldrich, Steinheim, Germany),
- dibutyl phosphite (DBPh; Sigma Aldrich, Steinheim, Germany),
- diphenyl phosphite (DPPh; Sigma Aldrich, Steinheim, Germany),
- dibutyl phosphate (DBP; Sigma Aldrich, Steinheim, Germany).

**Figure 1:**
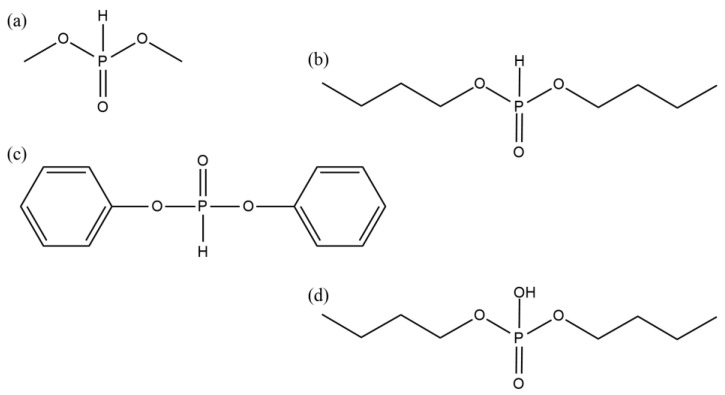
The chemical structures of phosphorus telogens: DMPh (**a**), DBPh (**b**), DPPh (**c**), DBP (**d**).

The chemical structures of the tested telogens are shown in Figure 1.

(c) bis(2,4,6-trimethylbenzoyl)-phenylphosphineoxide (APO; Omnirad 819, IGM Resins, Waalwijk, The Netherlands).

The varnish compositions were prepared using P-telomer syrups, pentaerythritol triacrylate (PETIA; Allnex, Drogenbos, Belgium) as a crosslinking monomer, and 1-hydroxycyclohexylphenyl ketone (HAP; Omnirad 184, IGM Resins, Waalwijk, The Netherlands) as radical UV-photoinitiator. The addition of PETIA cross-linking monomer was intended to increase the cross-linking density of the coating and potentially improve its properties.

### 2.2. P-Telomers Syrups and Coatings Preparation

The P-telomers syrups were prepared via UV-induced telomerization processes of mixtures compounded of BA, MMA, HEA, STY, radical UV-photoinitiator (APO), and one of four organophosphorus telogens (DMPh, DPPh, DBPh, or DBP). Reactions were realized in the presence of argon at 50 °C for 50 min in a glass reactor (250 mL) equipped with a mechanical stirrer, cooler, and thermocouple. The UV-LED stripe (λ = 390 ± 5 nm; MEiSSA, Warsaw, Poland) was used as a UV light source. The UV irradiation inside the reactor (15 mW/cm^2^) was controlled using the SL2W UV-radiometer (UV-Design, Brachttal, Germany).

Coating compositions were formed by mixing P-telomers syrups with the multifunctional crosslinking monomer (PETIA) and UV-photoinitiator (HAP). Compositions were stored for 24 h in a dark place, then applied onto glass substrates using a gap applicator (60 µm) and UV-irradiated (total UV dose of 6 J/cm^2^) by means of the medium-pressure mercury lamp (UV-ABC, Hönle UV-Technology, Gräfelfing, Germany). The systems used for P-telomers, and varnishes preparation are shown in Table 1.

### 2.3. Characterization of P-Telomers Syrups

The dynamic viscosity of the telomers syrups was determined with the DV-II Pro Extra viscometer (spindle #6 or #7, 50 rpm; Brookfield, New York, NY, USA), at 25 °C. The solid content of the syrups was measured by means of the thermobalance (Radwag, Warsaw, Poland); samples (ca. 2 g) were heated in aluminum pans at 105 °C for 4 h. Gel permeation chromatography (GPC) was used for the determination of the molecular masses (Mw, Mn) and polydispersity (PDI) of the P-telomers (telomers syrups were heated at 140 °C for 4 h before the test in order to remove unreacted monomers). The kinetics studies of the UV-induced telomerization process were realized at 50 °C by the photo-DSC method (the differential scanning calorimeter with UV equipment; Q100, TA Instruments, New Castle, DE, USA). Samples (of 5 mg) were UV-irradiated (320–390 nm) with an intensity of 15 mW/cm^2^ in an argon atmosphere.

During the measurements, the polymerization rate (*R_p_*, %/*s*), conversion of double bonds (*p*, %), and photoinitiation index (*I_p_*, *s*^−2^) were calculated according to the following equations:

(1) $$R_{p}=\frac{\left(\frac{dH}{dt}\right)}{H_{0}}\left[1/s\right]$$

(2) $$p=\frac{\Delta H_{t}}{\Delta H_{0}}\cdot 100\;\left[\%\right]$$

(3) $$I_{p\;}=\frac{R_{p}^{max}}{t_{max}}$$

where: *dH*/*dt* is the heat flow recorded during UV-irradiation, *H*_0_ is the theoretical heat value for the complete degree of conversion (Δ*H* = 78.0 kJ/mol for acrylates, Δ*H* = 54.0 kJ/mol for methacrylates and Δ*H* = 67.4 kJ/mol for styrene), Δ*Ht* is the reaction heat evolved at time *t* [37].

The infrared spectra, measured with the Fourier transform infrared spectroscope with ATR accessories (Nicolet 380, ThermoScientific, Waltham, MA, USA) were used to characterize the incorporation of groups derived from phosphorus telogens (DMPh, DBPh, DPPh, and DBP) in the structure of the dry telomers (prepared by drying the reaction mixtures at 140 °C for 4 h before the test to remove unreacted monomers).

### 2.4. Characterization of the Varnishes

Conversion of *C = C* bonds (*DC_C = C_*) in the varnish coatings after a UV-irradiation process was monitored using the Fourier transform infrared spectroscope with ATR accessories (Nicolet 380, ThermoScientific, Waltham, MA, USA) and calculated according to Equation (4):

(4) $$DC_{c=c}=\left(1-\frac{A\left(t\right)}{A\;\left(0\right)}\right)\cdot 100\;\left[\%\right]$$

where *A*(0) is the initial intensity of the peak at 1635 cm^−1^ and *A*(*t*) is the intensity of the peak at 1635 cm^−1^ after t-time. The mentioned peaks were normalized in relation to the reference peaks at ca. 2960 cm^−1^ (stretching vibration of asymmetric aliphatic -CH_2_- groups in the coating compositions) [38].

Selected mechanical properties of the cured coatings were determined. The pendulum hardness of varnishes cured on a glass substrate was tested using the König pendulum (AWS-5, Dozafil, Warsaw, Poland) according to the PN-EN ISO 1522 standard (four measurements for each sample). Adhesion of the coatings to the glass substrate was measured according to the ISO 4624 standard using a pull-off adhesion tester—Elcometer 510 (Elcometer, Manchester, UK), aluminum dollies (20 mm), and a two-component epoxy adhesive (results are average made from five measurements). Gloss (at 20°, ISO 2813) and smoothness (expressed by Distinctness of Image (DOI) parameter, ASTM D5767) of varnish coatings applied on white paper panels (WDX Plain White Cards, Leneta Company, Mahwah, NJ, USA) were measured using the IQ20/60/85 device (four measurements for each sample: Rhopoint Instruments, St. Leonards-on-Sea, UK). The scratch resistance test was performed using a scratch apparatus equipped with a steel scratch stylus (Dozafil, Warsaw, Poland) according to the PN-EN ISO 1518 standard. The presented results corresponded to the minimum load of the stylus causing a visible scratch on the surface of the coating. The solvent resistance of cured coatings was tested according to PN-EN ISO 2812-3 standard. During tests, cotton wool swabs soaked in acetone, propan-2-ol, or methyl ethyl ketone were placed on the surface of the coatings and covered with the watch glasses for 24 h. After that time, the swabs were removed, and test panels were wiped with dry cotton wool. The degree of destruction of the coatings was visually defined (according to ISO 4628-2 or ISO 4628-5). The water resistance of varnish coatings was determined according to the PN-76/C-81521 standard. Test samples were immersed in distilled water (20 ± 2 °C) for 24 h, dried with absorbent paper, and visually evaluated (according to ISO 4628-2).

## 3. Results and Discussion

### 3.1. Kinetics of UV-Phototelomerization Process

**Figure 2:**
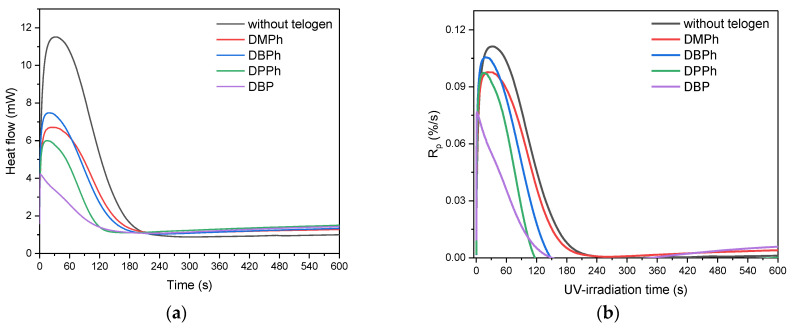

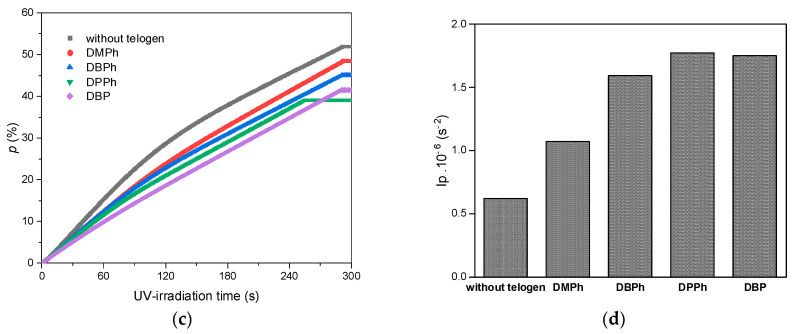
Photo-DSC curves (**a**), the reaction rate (**b**), double bond conversion (**c**), and photoinitiating index (**d**) during BA, MMA, HEA, and STY UV-phototelomerization process in the presence of different P-telogens (320–390 nm; I_o_ = 15 mW/cm^2^).

The results presented in the article refer for the first time to the photochemical telomerization of (meth)acrylate monomers and styrene in the presence of the APO photoinitiator and P-telogens with different chemical structures. At the beginning, the influence of the P-telogen’ structures on the process of UV-phototelomerization of the selected monomers (BA, MMA, HEA, STY) system was investigated by the photo-DSC method. The results of the kinetic studies of systems containing 0.006 moles of the telogen and 0.5 wt. part of APO (per 100 wt. parts of monomer mixtures) are presented in Figure 2.

Kinetic studies showed that the UV-photopolymerization of the tested monomers (without telogen) ran faster (*R_p_^max^* ca. 0.11%/s) than their UV-phototelomerization (with P-telogens). The maximum reaction rates (*R_p_^max^*) in UV-phototelomerization systems were arranged as follows: DBPh (0.10%/s) >DMPh and DPPh (0.09%/s) > DBP (0.07%/s). It is significant that in systems with P-telogen the time to reach the *R_p_^max^* was significantly shorter (from 1.2 s for a system with DBP, 18 s for DBPh, 19 s for DPPh, 24 s for DMPh) than during UV-photopolymerization (34 s). Thus, the ability to initiate reactions (*I_p_*, Figure 2d) in systems with P-telogens was significantly higher than in classical photopolymerization. In this respect, the system with DBP stood out. Dibutyl phosphate (DBP) is an ester of phosphoric acid, while the other tested telogens are derivatives of phosphonic acid. During the initiation of telomerization with DBP, photolysis of the P-OH bond took place, and in other cases, the P-H bonds were broken. The photo-DSC studies revealed that in the DBP-APO initiation system, the rapid formation of radicals was also accompanied by a lower maximum reaction rate. Upon reaching it, the reaction rate slowed down quite sharply. This might be due to the quenching of the excited states by collisions of hydroxyl radicals with macroradicals. Consequently, this system was characterized by the lowest double bond conversion value (p) after 5 min of UV irradiation (approx. 44%). The mixture without P-telogen was characterized by the highest value of monomer conversion (53%). Under the given reaction conditions (i.e., at 15 mW/ cm^2^ of UV-dose and APO concentration) monomers conversion was higher than approximately 50%. Among the samples with phosphonic acid esters (DMPh, DBPh, DPPh) there was a tendency that the larger the alkyl or aryl substituent, the higher the efficiency of reaction initiation (*I_p_*). The reaction also ended faster (rapid decrease of *R_p_* to zero), which means that the value of double bond conversion was lower.

### 3.2. Properties of P-Telomers and P-Telomers Syrups

**Figure 3:**
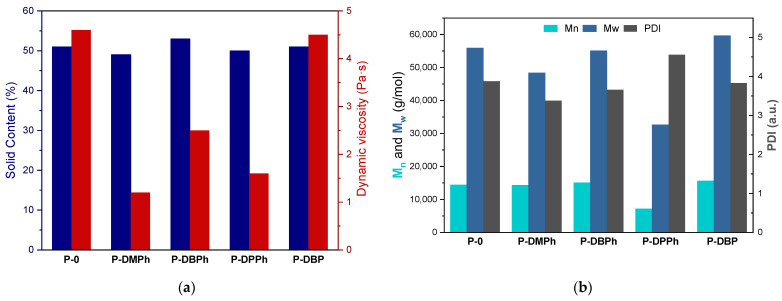
The values of dynamic viscosity and solid content of the P-telomers syrups (**a**), molecular weights (M_w_, M_n_), and polydispersity (PDI) of the P-telomers (**b**).

The UV-phototelomerization process in a glass reactor was conducted at the same intensity of UV radiation and temperature as the photo-DSC tests (15 mW/cm^2^ and 50 °C, respectively). The main difference was the mechanical mixing of the reactants during the process in a glass reactor. The products of this process were solutions of linear P-telomers (oligomers) in unreacted monomers, so-called P-telomers syrups. The aim was to achieve a similar monomer conversion in the syrups, i.e., the solids content (SC) of the telomers syrup. The selected physicochemical properties of the obtained P-telomers syrups (i.e., the solid content and dynamic viscosity), as well as molecular weights and polydispersity of dry telomers are presented in Figure 3. As mentioned above, the SC values were about 50% for each sample, independently of the type of phosphorus compound used in the process, which was most likely due to the amount of photoinitiator used. In contrast, the dynamic viscosity values of telomer syrups varied from 1.2 to 4.6 Pa·s. The highest value of dynamic viscosity was achieved for the reference sample P-0 (4.6 Pa·s), as expected. In general, the telomerization process leads to oligomers rather than polymers, hence the lower viscosity results of telomer syrups (1.2–2.5 Pa·s). The exception was P-DBP syrup, with a viscosity close to that of the reference sample P-0 (i.e., 4.5 Pa·s). Already in the photo-DSC studies, a slightly different course of the process with the participation of DBP telogen was revealed, i.e., very fast formation of reactive radicals. For this reason, longer chains of oligomers may be formed in the system, resulting in a higher viscosity of the telomer syrup. This is confirmed by the results of the molecular weight, presented in Figure 3b; for P-0 polymer and P-DBP telomer similar values of Mn and Mw (14,500 and 56,000 g/mol, respectively), as well as PDI values (3.8 a.u.) were observed. However, significantly lower Mn and Mw values were recorded for the P-DPPh telomer (7000 g/mol and 3300 g/mol, respectively), and PDI value was the highest (4.6 a.u.). The most favorable PDI results were recorded for the DMPh sample (3.3 a.u.). As it is known, organophosphorus compounds are not very effective chain transfer agents [36], which is proved by the presented research.

**Figure 4:**
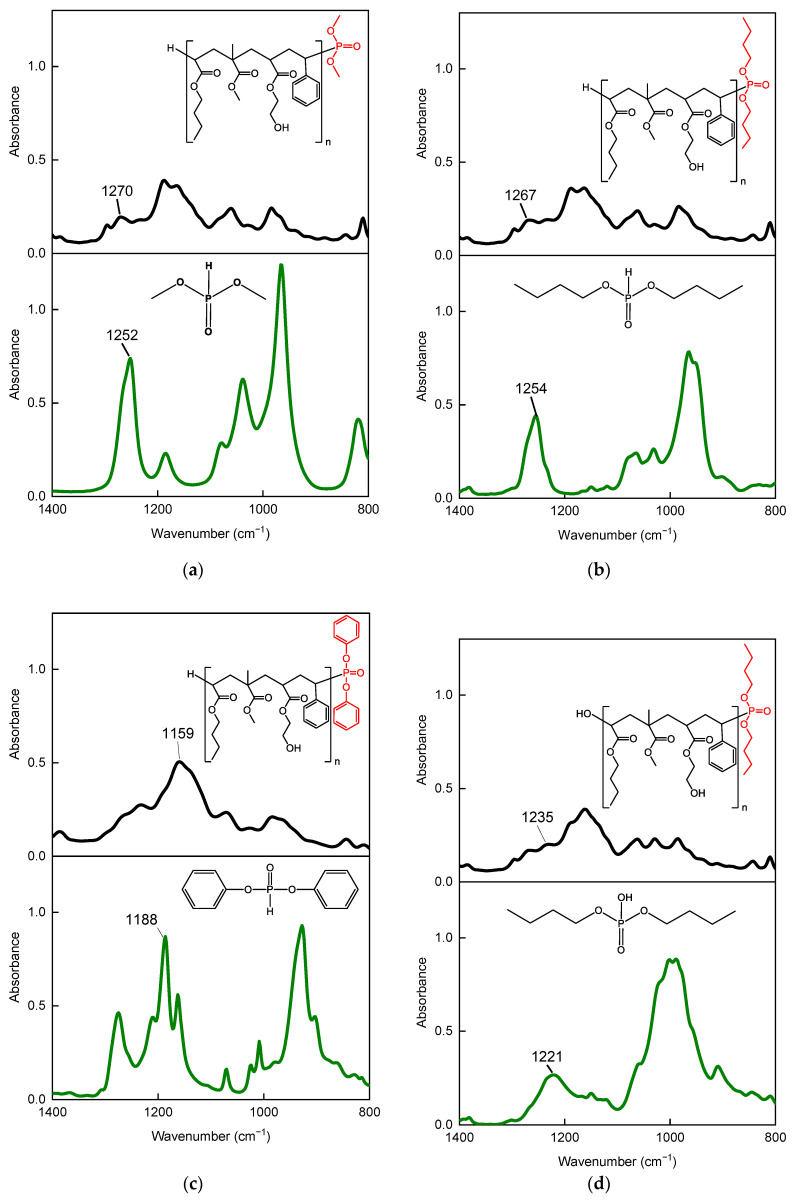
The FTIR spectra of: DMPh and P-DMPh telomer (**a**); DBPh and P-DBPh telomer (**b**); DPPh and P-DPPh telomer (**c**); DBP and P-DBP telomer (**d**).

The incorporation of groups derived from P- telogens (DMPh, DBPh, DPPh, and DBP) in the structure of the oligomers was checked by analyzing the FTIR spectra of the tested phosphorus compounds and dry telomers (Figure 4). The telogen spectra revealed peaks in the range 1220–1270 cm^−1^, attributed to P = O stretching vibrations in the telogen structures (DMPh, DBPh, and DBP), and at a wavelength of 1188 cm^−1^, derived from P-O-Ar stretching (DPPh). In the case of FTIR spectra of dry telomeres, a slight shift of the above-mentioned peaks (from 13 to 29 cm^−1^) was observed. It was probably caused by the presence in the structure of linear telomers of hanging hydroxyl groups derived from the HEA monomer [38]. Nevertheless, the FTIR results confirm the formation of phosphorus-containing oligomers, i.e., P-telomers. Their structures are also shown in Figure 4.

### 3.3. Properties of UV-Cured Varnish Coatings

Properties of the prepared UV-cured varnish coatings are influenced by many factors, i.e., the amount and structure of linear telomers, the number of unreacted monomers after the phototelomerization process, the type and amount of the crosslinking monomer or UV-photoinitiator added to the coating compositions (which participates in the photo-crosslinking process).

**Figure 5:**
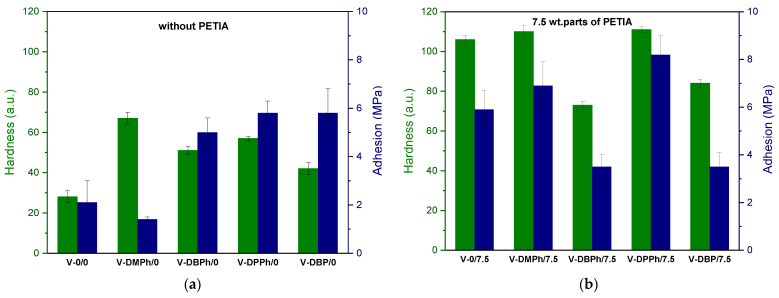
The values of hardness and adhesion of UV-cured varnish coatings: without crosslinking monomer PETIA (**a**), with 7.5 wt. parts of crosslinking monomer PETIA (**b**).

In this paper, the influence of the P-telomer type (i.e., the structure of terminal phosphorus groups) and the presence of crosslinking monomer on the mechanical and optical properties of UV-cured varnish coatings was tested. The hardness and adhesion of coatings (total UV dose 6 J/cm^2^) are presented in Figure 5.

**Figure 6:**
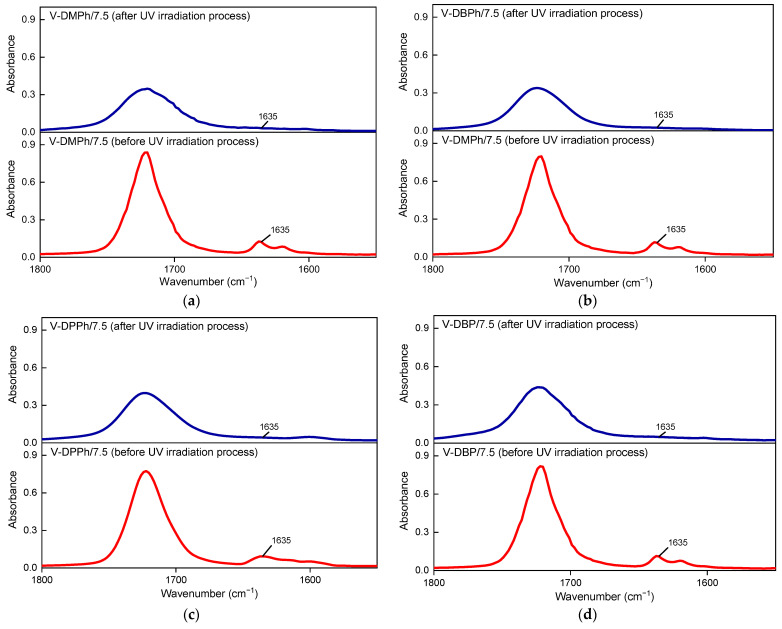
The FTIR spectra of UV-cured varnish coatings (before and after the UV irradiation process): V-DMPh/7.5 (**a**); V-DBPh/7.5 (**b**); V-DPPh/7.5 (**c**); V-DBP/7.5 (**d**).

As can be seen, coatings with crosslinking monomer PETIA (Figure 5b) generally showed higher hardness (80–110 a.u.) and adhesion to glass substrate (3.7–8.2 MPa) values than those without PETIA (30–75 a.u. and 1.7–6 MPa, respectively) (Figure 5a). That was due to the generally higher cross-linking density of the coatings containing PETIA and additionally the presence of hydroxyl groups (from the structure of this monomer). It should be noted that in PETIA systems, the UV-crosslinked coating consisted of a linear telomer (or poly(meth)acrylate; reference sample V-0/7.5) and a cross-linked structure composed of unreacted monomers (BA, MMA, HEA, STY) and the trifunctional PETIA monomer (semi-IPN). Therefore, the hardness and adhesion results were higher than those in Figure 5a. Excellent hardness values were observed for the samples V-DMPh/7.5 and V-DPPh/7.5 (ca. 110 a.u. and 111 a.u., respectively). These results were even higher than for the reference sample (V-0/7.5, 106 a.u.). These coatings were also characterized by higher adhesion to the glass substrate than the reference sample. The highest adhesion result was obtained for the V-DPPh/7.5 coating (8.2 MPa). Unexpectedly, the V-DBPh and V-DBP samples showed less adhesion to the substrate than the reference sample (ca. 3.8 MPa), and lower hardness values (79 a.u. and 82 a.u., respectively). Based on the FTIR spectra evaluation (Figure 6), no unsaturated bonds in the cured coatings were noted (*DC_C = C_* was 100%). On that basis, we believe that the lower adhesion of the V-DBPh/7.5 and V-DBP/7.5 coatings was due to the presence of relatively long aliphatic chains (C_4_) in the P-telomere structures, which created spatial obstacles, and the terminal groups containing phosphorus were trapped in the polymer matrix (semi-IPN), which resulted in deterioration of adhesion. Additionally, these coatings are based on telomeres with slightly higher molecular weights (which confirms the adverse effect on adhesion), and their syrups have the highest viscosity (which makes the photopolymerization process of the coating composition more difficult than in the others).

Regarding the results for coatings without PETIA (Figure 5a), samples based on P-telomers were allowed to achieve at least two times the higher hardness and adhesion values compared to the reference sample (V-0/0). However, it should be mentioned that the hardness values were unsatisfactory (<80 a.u.). The V-DMPh/0 coating was an exception here. The solution of this P-DMPh telomer was characterized by the lowest value of dynamic viscosity (1.2 Pa·s) (slightly molecular weight of oligomers, Figure 3b). Considering this and the comparable number of unreacted monomers in all P-telomers solutions, it should be stated that in the system with DMPh, the photopolymerization process of the remaining monomers was easier (due to the low viscosity of the telomer matrix). Therefore, it was easier to form polymer coils from linear telomers and linear poly(meth)acrylates, so that the access of phosphorus atoms to the glass surface was limited (they are trapped in polymer coils), which results in lower adhesion, but slightly higher hardness than other samples. The molecular weights of telomers also affected the adhesion results. The highest values were observed for the varnish coatings containing P-DPPh telomer (the shortest chains were noted). Short telomers chains are more mobile and allow better contact with the substrate surface (better wetting) and better ordering of the dipoles, so adhesion values are higher.

**Figure 7:**
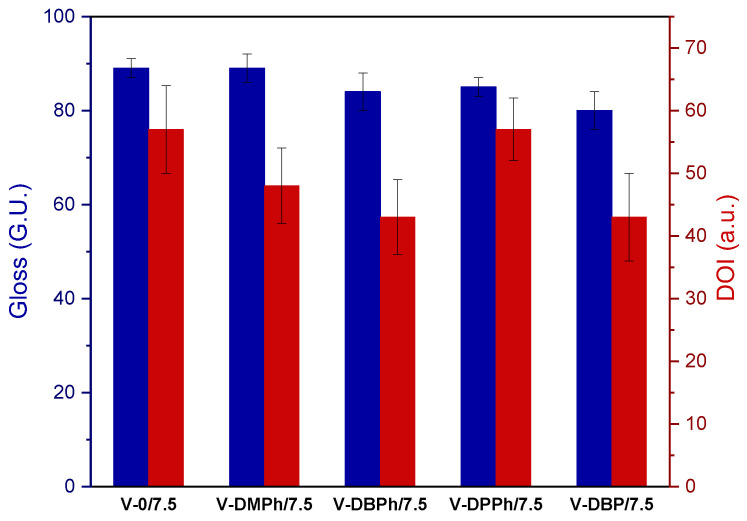
The values of gloss at 20° and DOI parameter of UV-cured varnish coatings containing crosslinking monomer.

In selected UV-cured varnish coatings containing a crosslinking monomer (total UV dose 6 J/cm^2^), the influence of the type of P-telomers on the gloss values (at 20°) was investigated. The prepared coatings were characterized by high gloss (from 80 to 89 G.U.) (Figure 7). The results were similar for all samples, regardless of the presence and type of P-telomer. It might therefore be concluded that the monomer composition in the tested systems, in particular the presence of styrene, was responsible for the high results. More significant differences were noted during measurements of the DOI parameter (described as the sharpness or clarity of the image produced by the reflection of an object on a surface varnish coating, the higher the DOI value the varnish coatings are smoother and clearer). Samples with P-DBPh and P-DBP telomers had the same DOI values (43 a.u.), but an increase of that parameter was observed for the varnishes based on P-DMPh and P-DPPh telomers (48 and 57 a.u., respectively). Additionally, the DOI of the reference sample was also 57 a.u. Generally, slight imperfections of tested varnish coatings were observed.

Resistance to scratch, the organic solvents, and distilled water were evaluated for UV-cured varnish coatings containing PETIA monomer (Table 2). The minimum load at which the stylus caused a visible scratch on the surface of the coating was noted. The values of that parameter were reported from 100 g to 350 g. Therefore, the coatings did not withstand impact scratch tests. However, among the tested samples, the highest scratch result was obtained for the varnish coating with P-DMPh telomers (scratch resistance the same as reference samples). Additionally, the V-DMPh/7.5 coating was more resistant to water than the other samples (including the reference sample). Moreover, V-DMPh coatings were characterized by slightly better resistance to organic solvents. On the other hand, the most significant degradation was observed in the case V-DBP/7.5 sample (cracking of the coating).

## 4. Conclusions

Phosphorus-containing (meth)acrylate telomers (P-telomers) have been prepared via a solvent-free UV-phototelomerization process in the presence of APO photoinitiator and different phosphorus telogens (phosphite or phosphate esters). The prepared P-telomers syrups compounded with the crosslinking monomer (pentaerythritol triacrylate; PETIA) and a UV-photoinitiator were tested as solvent-free UV-curable varnish liquid compositions. The influence of the structure of phosphorus telogens on the course of the UV-phototelomerization process and the influence of the structure of obtained P-telomers on the properties of UV-cured varnish coatings were characterized. The main results are as follows:

- P-telogen/APO initiating systems showed higher efficiency in the process than the APO photoinitiator itself (higher photoinitiation index), which resulted in a lower rate of telomerization reaction than in the case of photopolymerization and a slightly lower monomer conversion.
- Chemical modification of P-telomer syrups with a crosslinking monomer significantly improved the hardness and adhesion of the obtained coatings. The highest hardness values were reported for UV-cured varnish coating with dimethyl phosphite (DMPh) telomers (111 a.u.), while the strongest adhesive bond to the glass surface was observed in the case of diphenyl phosphite (DPPh) telomers (8.2 MPa).
- The most satisfactory scratch, water, and solvent resistance characterized UV-cured varnish coatings based on DMPh telomers.

## Figures and Tables

**Table 1:** Composition of P-telomers syrups and UV-photocurable varnishes.

P-Telomers Syrup	Comonomers [wt%]				Telogen			APO ^b^	Varnish Symbol	PETIA ^c^	HAP ^c^
	BA	MMA	HEA	STY	Symbol	Dose ^a^ (mole)	Dose ^b^ (wt. Parts)				
P-0	40	35	15	10	-	0	0	0.75	V-0/0	0	1
									V-0/7.5	7.5	
P-DMPh					DMPh	0.006	1.65		V-DMPh/0	0	
									V-DMPh/7.5	7.5	
P-DBPh					DBPh		2.90		V-DBPh/0	0	
									V-DBPh/7.5	7.5	
P-DPPh					DPPh		3.50		V-DPPh/0	0	
									V-DPPh/7.5	7.5	
P-DBP					DBP		3.15		V-DBP/0	0	
									V-DBP/7.5	7.5	

^a^—mole of the telogen/100 wt. parts of the monomers mixture. ^b^—wt. part/100 wt. parts of the monomers mixture. ^c^—wt. parts/100 wt. parts of the syrup.

**Table 2:** Scratch, water, and solvent resistance of the UV-cured varnish coatings.

Varnish Symbol	Scratch Resistance ^1^ (g)	Effect of Distilled Water ^2^	Effect of Organic Solvents ^2^		
			Acetone	Propan-2-ol	MEK
V-0/7.5	350	Blisters 5(S2)	Blisters 4(S3)	Blisters 4(S3)	Blisters 4(S3)
V-DMPh/7.5	350	Blisters 4(S2)	Blisters 5(S2)	Blisters 3(S2)	Blisters 5(S2)
V-DBPh/7.5	200	Blisters 5(S2)	Blisters 5(S3)	Blisters 5(S3)	Blisters 5(S3)
V-DPPh/7.5	100	Matt coating, blisters 5(S2)	Blisters 4(S3)	Blisters 4(S3)	Blisters 4(S3)
V-DBP/7.5	300	Matt coating, blisters 5(S2)	Cracking without preferential direction 5(S4)		

^1^—measured as the minimum load caused a visible scratch on the surface of the coating; ^2^—quantity and size of the blisters or cracking were characterized from 2 to 5 (the number “2” is the lowest value of the parameter), the first number is the quantity, and the second number (after “S”) is the size (according to ISO 4628-2 and ISO 4628-5).

## Data Availability

The data presented in this study are available on request from the corresponding author.
